# Sorting centimetre-long single-walled carbon nanotubes

**DOI:** 10.1038/srep30836

**Published:** 2016-08-01

**Authors:** Woo Jong Yu, Sang Hoon Chae, Quoc An Vu, Young Hee Lee

**Affiliations:** 1 Department of Electronic and Electrical Engineering, Sungkyunkwan University, Suwon 16419, South Korea; 2 Samsung-SKKU Graphene Center (SSGC), Sungkyunkwan University, Suwon 16419, South Korea; 3 Center for Integrated Nanostructure Physics, Institute for Basic Science (IBS), Suwon 16419, South Korea; 4 Department of Energy Science, Sungkyunkwan University, Suwon 16419, South Korea; 5 Department of Physics, Sungkyunkwan University, Suwon 16419, South Korea

## Abstract

While several approaches have been developed for sorting metallic (m) or semiconducting (s) single-walled carbon nanotubes (SWCNTs), the length of SWCNTs is limited within a micrometer, which restricts excellent electrical performances of SWCNTs for macro-scale applications. Here, we demonstrate a simple sorting method of centimetre-long aligned m- and s-SWCNTs. Ni particles were selectively and uniformly coated along the 1-cm-long m-SWCNTs by applying positive gate bias during electrochemical deposition with continuous electrolyte injection. To sort s-SWCNTs, the Ni coating was oxidized to form insulator outer for blocking of current flow through inner m-SWCNTs. Sorting of m-SWCNTs were demonstrated by selective etching of s-SWCNTs via oxygen plasma, while the protected m-SWCNTs by Ni coating remained intact. The series of source-drain pairs were patterned along the 1-cm-long sorted SWCNTs, which confirmed high on/off ratio of 10^4^–10^8^ for s-SWCNTs and nearly 1 for m-SWCNTs.

Because of excellent electrical properties of single-walled carbon nanotubes (SWCNTs) such as extremely high intrinsic carrier mobility with ballistic electron transport, high current-carrying capacity, and nanometer-sized low-dimensional structure^1^,^2^,^3^,^4^,^5^,^6^, together with their matured process technologies over other materials, SWCNTs are expected as one of the primary candidates for future electronics and optical device materials^9^. Although carbon nanotube computer was recently demonstrated utilizes aligned SWCNTs^9^, challenges still exist for macro-scale application of SWCNTs. One significant challenge is a mixture of metallic (m) and semiconducting (s) SWCNTs. Numerous techniques have been developed for sorting of m-SWCNTs and s-SWCNTs; electrical breakdown^16^, dynamic supramolecular coordination chemistry^17^, H-bonded supramolecular polymer^18^,^19^, selective etching by gas-phase reaction^20^, light irradiation^21^, dielectrophoresis^22^, DNA-assisted dispersion and separation^23^, ultra-centrifugation-based separation^24^, selective chemical functionalization^25^,^26^, and thermocapillary flows^27^,^28^. However, these approaches are limited in micrometer-long or smaller SWCNTs, which restrict excellent electrical performances of SWCNTs for macro-scale applications. In particular, integrated circuit has been demonstrated using individual SWCNTs, which length was limited in 18-μm-long^29^. In this work, we demonstrated a sorting method of centimetre-long m-SWCNTs and s-SWCNTs from the horizontally aligned SWCNT array on substrate synthesized by chemical vapor deposition (CVD)^30^.

## Results

**Figure 1:**
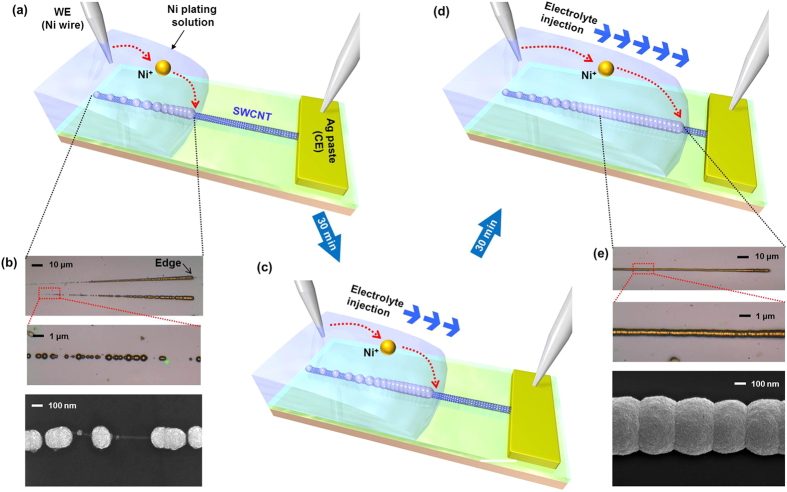
I Uniform Nickel coating on centemetre-long SWCNT. (**a**) Schematic illustration and optical image of Ni deposition on centemetre-long SWCNT without injection of Ni electrolyte. (**b**) Optical images and SEM image of Ni-deposited SWCNT without injection of Ni electrolyte. (**c**,**d**) Schematic illustrations and optical images of Ni deposition with continuous injection of Ni electrolyte by syringe pump. (**e**) Optical images and SEM image of Ni-coated SWCNT with continuous injection of Ni electrolyte.

Figure 1 illustrates the schematic of how to coat Ni particles uniformly along the centimetre-long SWCNTs. Electrochemical Ni deposition on the SWCNTs was carried out by flowing current between working electrode (WE, Ni wire) and counter electrode (CE, silver paste)^31^,^32^,^33^. Note that the Ni atoms are selectively nucleated at the defect sites with high chemical reactivity during electrochemical deposition^32^. As for a SWCNT, the resistivity cannot be ignored because of its one-dimensional nature. The resistivity 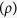 of an SWCNT is usually described by the electron mean free path (*l*) for backscattering. At the SWCNT length *L* < *l*, the transport is essentially ballistic and there is no resistivity decrease. The resistivity starts to scale linearly with *L* at *L* > *l* because of the backscattering of electrons. In our system, the resistivity of SWCNT scales linearly with *L* since *L* (>1 cm) is longer than *l* (1 μm^34^,^35^). Therefore, the resistance of SWCNT is lowest at the edge of electrolyte solution with respect to CE and gradually increases with the SWCNT located at the inner side of electrolyte solution. Consequently, the Ni particle density on the SWCNT is the highest at the edge of electrolyte solution to form continuous Ni coating and decreases exponentially as the distance is away from the CE electrode, forming discrete Ni islands in the region of electrolyte solution (Fig. 1a,b).

**Figure 2:**
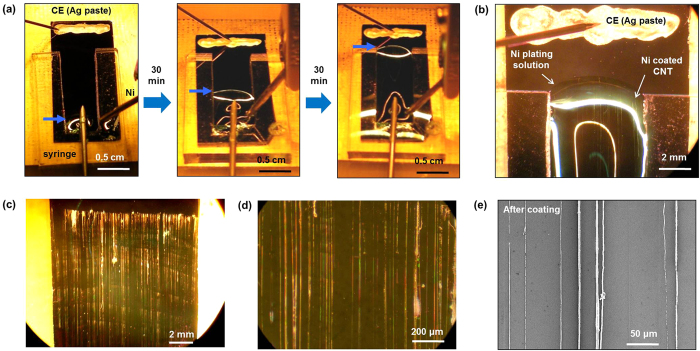
I Optical and SEM images of Ni coated SWCNT array. (**a**) Optical images of electrochemical Ni deposition on centmetre-long SWCNT with continuous injection of electrolyte solution using syringe and syringe pump. Edge of Ni electrolyte solution (blue arrow) is continuously shifts toward counter electrode as time goes on. (**b**) Optical image near the edge of Ni electrolyte solution during Ni deposition. White lines inside of electrolyte are Ni-coated SWCNT array. (**c**) Optical image of Ni-coated SWCNT array after Ni deposition. (**d**) Magnified optical image of (**c**,**e**) SEM image of Ni-coated SWCNT array.

For a uniform Ni coating over centimetre-long SWCNT, the edge of electrolyte solution was continuously shifted along the SWCNT (Fig. 1c,d, Fig. 2a) with continuous injection of electrolyte solution using a syringe pump (Supplementary information S1). The continuous Ni coating was followed electrolyte edge movement, resulting in uniform Ni coating along the centimetre-long SWCNT (Fig. 1e). Figure 2 shows optical and scanning electron microscope (SEM) images of uniformly Ni-coated SWCNT array by continuous injection of Ni electrolyte solution (Additional sample images of Ni-coated SWCNT array are shown in Supplementary information S2). It is note worthy that the Ni thickness is different for SWCNTs due to the resistance difference^29^ (Fig. 1i, Supplementary information 3). The Ni uniformity can be increased by reducing electrolyte injection rate (Supplementary information S4).

**Figure 3:**
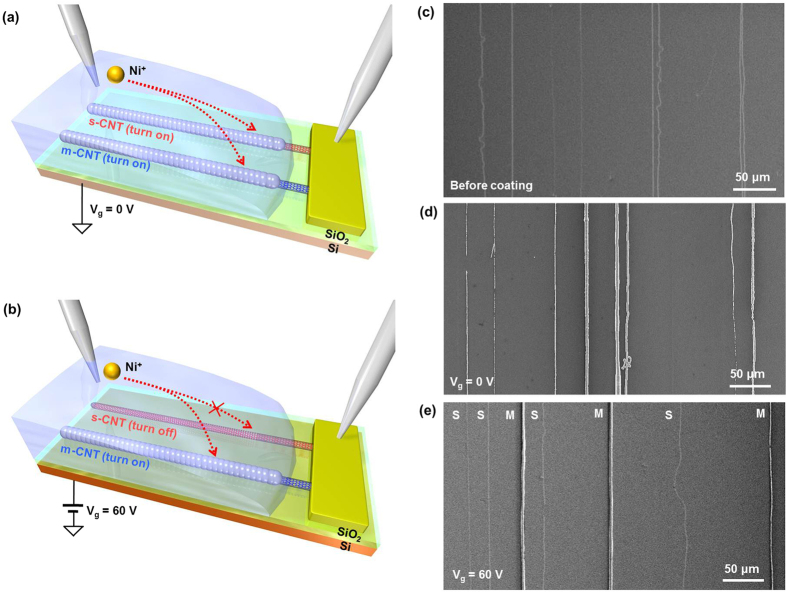
I Identifying s- and m-SWCNTs by selective Ni deposition on m-SWCNTs. (**a**) Schematic illustration of Ni deposition on s-SWCNT and m-SWCNT without backgate bias. (**b**) Schematic illustration of selective Ni deposition on m-SWCNT by applying positive backgate bias. (**c**) SEM image of SWCNT array before Ni deposition. (**d**) SEM image of Ni-coated s-SWCNTs and m-SWCNTs after Ni coating with V_g_ = 0 V. (**e**) SEM image of Ni-coated m-SWCNTs and bare s-SWCNTs after selective Ni coating with V_g_ = 60 V. ‘S’ is s-SWCNT and ‘M’ is m-SWCNT with Ni coating.

We further investigated the selective coating of Ni particles by applying backgate bias during Ni deposition (Fig. 3). Without gate bias application, both m-SWCNTs and s-SWCNTs allowed current flow during the Ni deposition, resulting in Ni coating on both SWCNTs (Fig. 3a). Selective Ni coating on m-SWCNTs can be demonstrated by applying positive gate voltage (V_g_ = 60 V) during Ni deposition (Fig. 3b). Only m-SWCNTs can flow current at positive gate voltage, while p-type s-SWCNTs are electrically turned off^S^. Figure 2c–e show SEM images of the SWCNT array before (Fig. 3c) and after Ni coating with a gate bias of 0 V (Fig. 3d) and 60 V (Fig. 3e). Before coating, bare SWCNTs are shown as blurred lines before Ni coating in Fig. 3c. Raman spectrum of uncoated (semiconducting) SWCNT is shown in supplementary information S5. All SWCNTs in Fig. 3d are shown as white lines due to the Ni coating on both s-SWCNTs and m-SWCNTs, while only m-SWCNTs are shown as white lines with distinguishably blurred s-SWCNTs (Fig. 3e).

**Figure 4:**
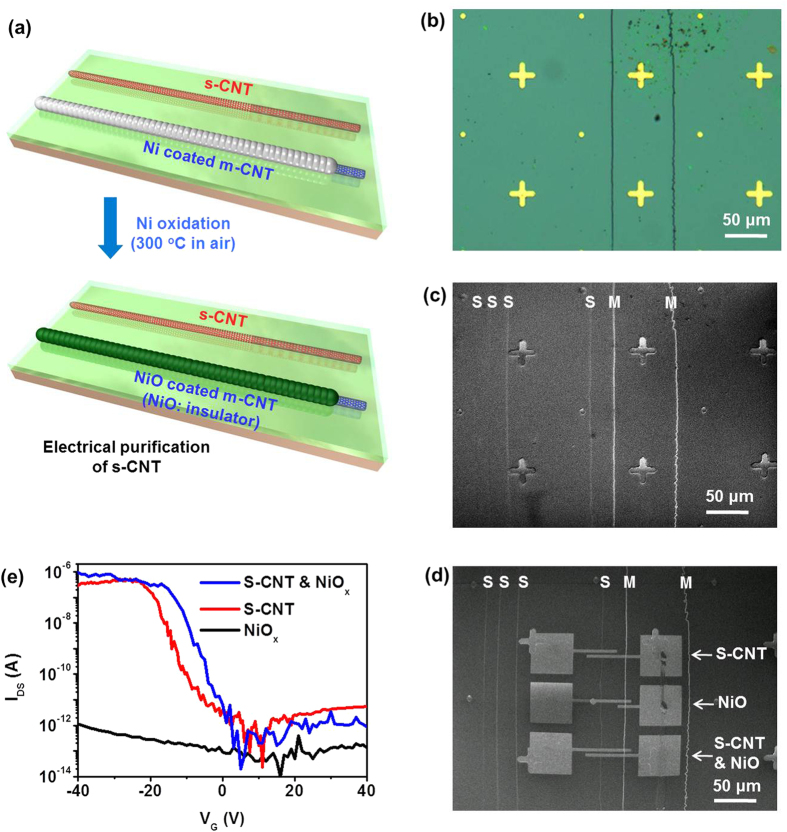
I Sorting s-SWCNT. (**a**) Schematic illustration of sorting of s-SWCNT by oxidation of Ni coating on the m-SWCNT. (**b**) Optical image of SWCNT array after Ni oxidation. Dark green lines are oxidized Ni coating on the m-SWCNTs. (**c**) SEM image of (**b**). Uncoated s-SWCNTs and NiO-coated m-SWCNTs are indicated with ‘S’ and ‘M’, respectively. (**d**) SEM image of the same area as (**c**) with three pairs of source-drain electrodes for electrical characteristics of s-SWCNT (top), NiO-coated m-SWCNT (middle), and s-SWCNT with NiO-coated m-SWCNT (bottom). (**e**) Transfer characteristics of s-SWCNT (red), NiO-coated m-SWCNT (black), and both s-SWCNT & NiO-coated m-SWCNT (blue) in (**d)**.

To sort out s-SWCNTs, the selective Ni coated sample was further baked at 300 °C for an hour under ambient conditions (Fig. 4a). The Ni particles coated on the m-SWCNTs were oxidized and turned into NiO, which is an insulator at room temperature with a resistivity well exceeding ~10^6^ Ω cm with a wide bandgap of 3.6–4.0 eV^36^. NiO outer shell can prohibit the current flow on inner shell m-SWCNTs when the contact source/drain electrode is constructed on top of NiO. Figure 4b,c shows the optical and SEM images of SWCNTs array after oxidation. The uncoated s-SWCNTs were not visible in optical image (Fig. 4b) but visible in SEM image at an accelerating voltage of 1 kV (Fig. 4c). Meanwhile, NiO coated m-SWCNTs were observed in both optical and SEM images. We patterned three pairs of source and drain electrodes to connect the s-SWCNT, NiO-coated m-SWCNT, and both s-SWCNT & NiO-coated m-SWCNT to measure electrical characteristics (Fig. 4d). In the transfer characteristics (Fig. 4e), s-SWCNT shows typical p-type characteristics with a high on/off current ratio of ~10^6^. Meanwhile, a negligible current of a few pA was observed on NiO-coated m-SWCNT, indicating that the NiO_x_ clearly works as an insulating layer. The FET on both s-SWCNT & NiO-coated m-SWCNT also shows a clear semiconducting behavior, demonstrating electrically sorted s-SWCNTs.

**Figure 5:**
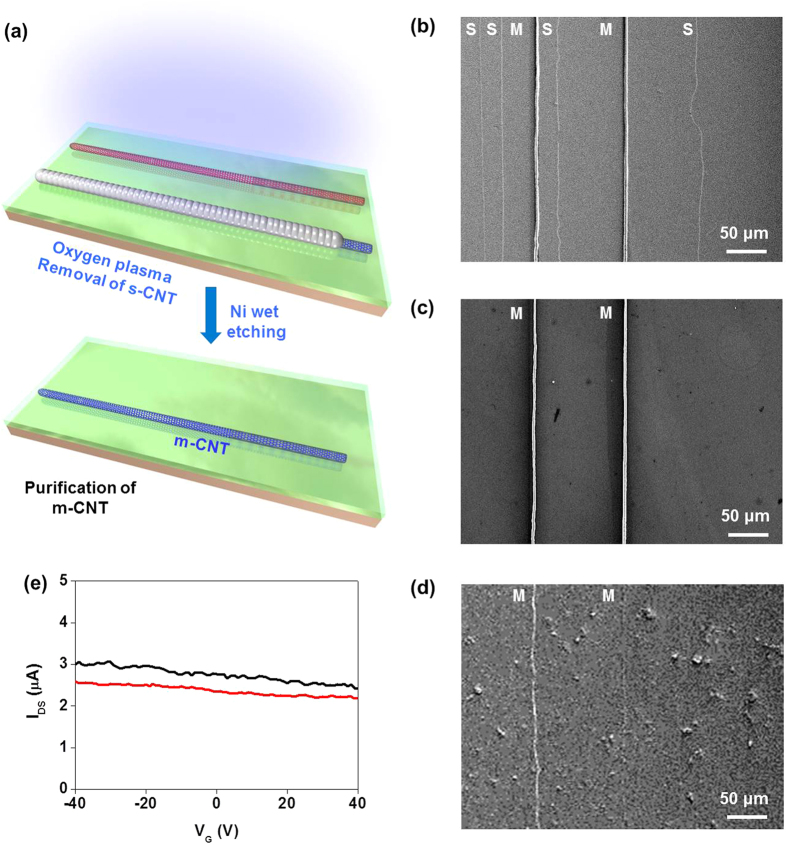
I Sorting m-SWCNT. (**a**) Schematic illustration of sorting of m-SWCNT by removal of bare s-SWCNT by oxygen plasma etching. Coated Ni protects the m-SWCNT from oxygen plasma. Only m-SWCNT array remained on the substrate after dissolving the Ni coating. (**b**) SEM image of selectively Ni-coated m-SWCNTs and bare s-SWCNTs. (**c**) SEM image after selective removal of s-SWCNTs by oxygen plasma etching. (**d**) SEM image of m-SWCNTs after Ni dissolution. (**e**) Transfer characteristics of sorted m-SWCNTs.

The sorting of m-SWCNTs were demonstrated by selectively etching s-SWCNTs by oxygen plasma treatment on selective Ni coated sample (Fig. 5a). The m-SWCNTs were protected by the Ni particles during oxygen plasma treatment, while bare s-SWCNTs were etched away. The pure m-SWCNTs array was obtained by dissolving Ni particles in Ni etchant after plasma treatment. Figure 5b,c show SEM images of Ni-coated m-SWCNTs array before and after oxygen plasma treatment, respectively. The s-SWCNTs clearly disappeared after oxygen plasma etching, while Ni-coated m-SWCNTs still remained intact (Fig. 5c, Supplementary information S6). Bare m-SWCNTs were visible in Fig. 5d after dissolving the Ni particles in Ni etchant (Fig. 5d, Supplementary information S6). The transfer characteristics clearly show the metallic behavior without gate dependence (Fig. 5e). Note that some of Ni residue particles may remain on the surface of m-SWCNT. The Ni particles on m-SWCNTs, however, can enhance the conductivity and sensitivity of m-SWCNT^37^.

**Figure 6:**
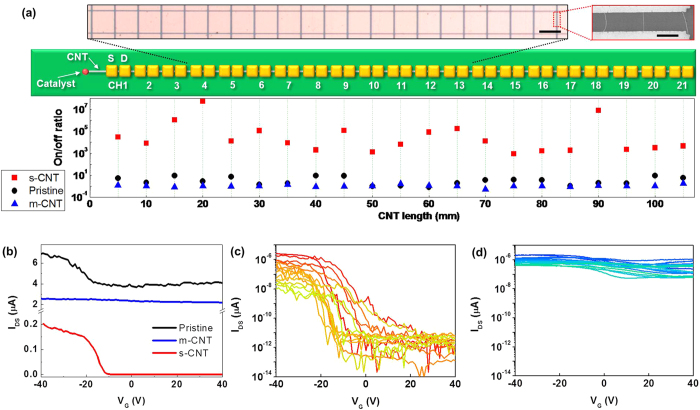
I Electrical characteristics of sorted semiconducting and metallic SWCNT along the 1-cm-long SWCNT. (**a**) Optical, SEM images and schematic illustration of 21 FET array along the 1-cm-long SWCNT (top) and their on/off ratio (bottom). Scale bar: 250 μm (optical), 10 μm (SEM). (**b**) Transfer characteristics before sorting (black line), after sorting of m-SWCNT (blue line) and s-SWCNT (red line). Transfer characteristics of 21 FETs (**c)** along the 1-cm-long sorted s-SWCNT and (**d**), sorted m-SWCNT.

The significance of our sorting method is to obtain aligned centimetre-long m-SWCNTs and s-SWCNTs directly from substrate, which is distinguished from conventional powder approaches. Furthermore, selective Ni coating on m-SWCNTs and simple oxidation without sophisticated electrical burning steps is a big advantage from integration point of view. To demonstrate it, we fabricated 21 source-drain pairs along the 1-cm-long m-SWCNT and s-SWCNT (electrode width of 250 μm and channel length of 10 μm) (Fig. 6a) (Supplementary information S7). About 3~10 SWCNTs channels were contained in each source-drain pairs. Figure 6b shows transfer characteristics of pristine sample (black line), sorted m-SWCNT (blue line) and sorted s-SWCNT (red line). In the pristine sample, m-SWCNTs and s-SWCNTs were mixed among the 3~10 SWCNTs channels in each FET with a presumable theoretical ratio of 1:2. Transfer characteristics of the pristine sample showed a small gate modulation (black line in Fig. 6b) due to the presence of s-SWCNT. However, high off-current from m-SWCNTs limits the on/off ratio lower than 10. Meanwhile, the sorted s-SWCNT shows large gate modulation with an average on/off ratio of >10^4^ (red line in Fig. 6b) and sorted m-SWCNT shows almost no gate dependence with an on/off ratio nearly 1 (blue line in Fig. 6b). The on/off ratio of 21 pairs of CNT-FETs along the 1-cm-long SWCNT (Fig. 6a) and their transfer characteristics (Fig. 6c,d) indicating that 1-cm-long SWCNT channel maintained the semiconducting or metallic behaviour^38^.

## Discussion

The sorting method introduced here provides scalable and efficient means for centimetre-long aligned m- and s-SWCNTs. The series of source-drain pairs along the 1-cm-long sorted SWCNTs showed a high on/off ratio of 10^4^–10^8^ for s-SWCNTs and nearly 1 for m-SWCNTs. In the on/off ratio investigation along the rows of electrodes array (Fig. S8), the sorted m-SWCNTs showed lower on/off ratio than that of pristine sample in the all columns, indicating complete elimination of s-SWCNTs by oxygen plasma etching. The on/off ratios in sorted s-SWCNT array were about 10^2^~10^5^ times higher than pristine samples in overall rows, except few rows shown on/off ratios of lower than 10 (Fig. S8). It is suspected that few m-SWCNTs are partially uncovered with NiO_x_. The mixture of at least single m-SWCNT out of 3~10 SWCNT channels in each source-drain pairs can significantly reduce the on/off ratio. More careful Ni deposition with lower injection speed (Fig. S4) could demonstrate the complete sorting of s-SWCNTs. It should be noted that the aligned ultra-long SWCNTs prepared on silicon substrates are advantageous because the processing steps of the commercial digital electronics are fully compatible. Our method can be adopted for future large-scale integration with nano-scale individual CNTs as a conductive metal wire using metallic CNTs and a semiconductor switching channel using semiconducting CNTs, which is very encouraging for future high-speed digital electronics due to ballistic transport in individual SWCNTs.

## Methods

### Synthesis of ultralong SWCNTs

1-cm-long SWCNT array was synthesized by catalytic CVD using ethanol as the carbon source and FeCl_3_ or CoCl_2_ as a catalyst precursor^30^. To start, a 0.01 M catalyst solution was applied to one edge of a Si substrate by micro-contact printing. The substrate with a catalyst precursor was then placed in a horizontal 4 cm quartz tube furnace with the catalyst end facing the gas flow. The catalyst precursor was reduced in flowing Ar/H_2_ (500 sccm/30 sccm) gas mixture at 950 °C for 30 minutes and then ethanol vapor was introduced into the furnace by bubbling 200 sccm Ar through ethanol solution for growth of centimetre-long SWCNT. At the end of the synthesis, the reactor was purged with Ar/H_2_ (500 sccm/30 sccm) and cooled to room temperature.

### Deposition of Ni particles in Ni electrolyte

Conductive silver paste was formed on the surface of the prepared SWCNT array on SiO_2_(500 nm)/Si wafer as a counter electrode (CE) for electrochemical deposition^39^. Ni electrolyte solution with 270 g/L of NiSO_4_·6 H_2_O and 40 g/L of H_3_BO_3_ in deionized water was used for electrochemical Ni deposition. Ni wire was used for working electrode (WE) for continuous supply of Ni^+^ ions. Ni deposition was performed by flowing current between WE and CE with applied voltage of 5 V. For the selective Ni coating on m-SWCNT, a backgate voltage of 60 V was applied.

### Fabrication and characterization of CNT-FETs

The source and drain were patterned by photolithography. The metals of Cr (5 nm)/Au (60 nm) were deposited by an e-beam evaporator at 2 × 10^−6^ Torr. The photoresist was lifted off with metals and the remaining metals were used for source and drain electrodes. The electrical characteristics were measured under ambient conditions by source measure units (Keithley 236, 237) using a probe station. Scanning electron microscope (JEOL, JSM-7401F) images were taken by secondary electron image mode under a pressure of 4 × 10^−3^ Torr.

## Additional Information

**How to cite this article**: Yu, W. J. *et al*. Sorting centimetre-long single-walled carbon nanotubes. *Sci. Rep.* **6**, 30836; doi: 10.1038/srep30836 (2016).

## Supplementary Material

Supplementary Information
