# Supplementary material for: Sorting centimetre-long single-walled carbon nanotubes
Source: Sci Rep. 2016 Aug 1;6:30836. doi: 10.1038/srep30836 (PMC4967858; doi:10.1038/srep30836)
Supplement: Supplementary Information [file srep30836-s1.doc]

Supplementary information

Sorting centimetre-long single-walled carbon nanotubes

Woo Jong Yu^*^, Sang Hoon Chae, Quoc An Vu, Young Hee Lee^*^

**Supplementary Information**

S1. Experimental set up for uniform Ni coating on centimetre-long SWCNT array by electrochemical deposition.

S2. Uniform Ni coated SWCNT array.

S3. Ni coverage on SWCNTs depends on the resistance of SWCNT.

S4. Ni coverage on SWCNT depends on injection speed of Ni electrolyte solution.

S5. [Raman spectrum](javascript:popupOBO('CMO:0000823','C2NR00043A')) of uncoated (semiconducting) SWCNT

S6. SEM images before and after Ni removal on m-SWCNT.

S7. Source-drain pairs array for electrical characterization of sorted semiconducting and metallic SWCNT along the 1-cm-long SWCNT.

S8. On/off ratios of before and after sorting SWCNTs along the rows of electrodes array.

**S1. Experimental set up for uniform Ni coating on centimetre-long SWCNT array by electrochemical deposition.**

**Figure S1I a** and **b,** Optical image of electrochemical Ni deposition on centimeter SWCNT array with continuous electrolyte solution injection using syringe and syringe pump.

Syringe and syringe pump was used for continuous electrolyte solution injection (Fig. S1a). Centemetre-long SWCNT array grown silicon wafer was placed on the plastic board with gold gate-pad. Silicon back gate and gold gate-pad were electrically connected by conducting silver paste for gate bias application (Fig. S1b). Conductive silver paste on the top side of silicon wafer (head of SWCNT array) was used as counter electrode (CE) to flow the current on SWCNT array. The PDMS mold was placed on the silicon wafer to guide the injected electrolyte solution. Ni electrolyte solution was fully isolated to the silicon back gate by silicon oxide layer (500 nm) and PDMS fluidic channel to prevent gate leakage current flow. Ni wire was used as working electrode (WE) for continues Ni ion supply. Ni wire was dipped into the Ni electrolyte solution and bias voltage was applied between Ni wire (WE) and silver pates (CE). Ni ions in electrolyte solution are attracted and coated on SWCNTs array with current flow between WE and CE.

**S2. Uniform Ni coated SWCNT array.**

**Figure S2I** Scanning electron microscope (SEM) of with **a,** high density, **b,** low density, and **c,** turbulence SWCNT array, **d-i,** and their optical images after Ni coating.

**S3. Ni coverage on SWCNTs depends on the resistance of SWCNT.**

**Figure S3I** SEM image of Ni coated SWCNTs with various resistance SWCNTs, respectively at the electrolyte injection speed of 0.5 ml/h. 5 V was applied between CE and WE for electrochemical deposition. Ni particles on high-resistance SWCNTs are discrete with fast injection speed.

**S4. Ni coverage on SWCNT depends on injection speed of Ni electrolyte solution.**

**Figure S4I** SEM image of Ni coated SWCNTs (high resistance) with electrolyte solution injection speed of **a,** 0.5 ml/h **b,** 0.25 ml/h and **c,** 0.12 ml/h. V_ds_ = 5 V was applied. Lower injection speed increase Ni deposition time at the edge of electrolyte, resulting in a thick and uniform Ni coating on high resistance SWCNTs.

**S5.** [**Raman spectrum**](javascript:popupOBO('CMO:0000823','C2NR00043A'))**of uncoated (semiconducting) SWCNT.**


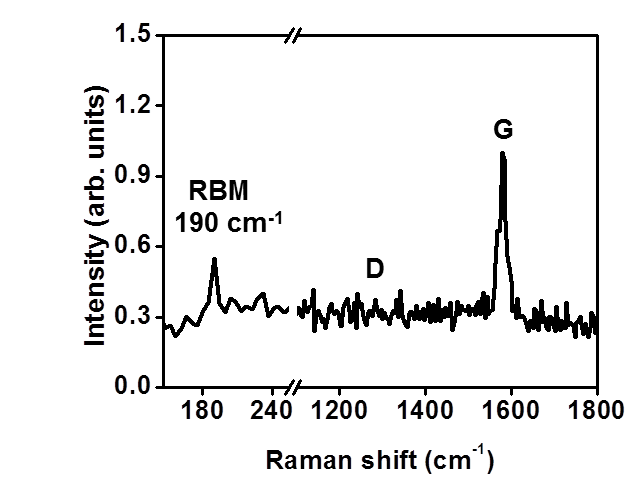


**Figure S5I** [Raman spectrum](javascript:popupOBO('CMO:0000823','C2NR00043A')) of uncoated (semiconducting) SWCNT with an excitation wavelength of 514 nm. The RBM peak was shown at 190 cm^-1^, indicating the semiconductor SWCNT.

**S6. SEM images before and after Ni removal on m-SWCNT.**


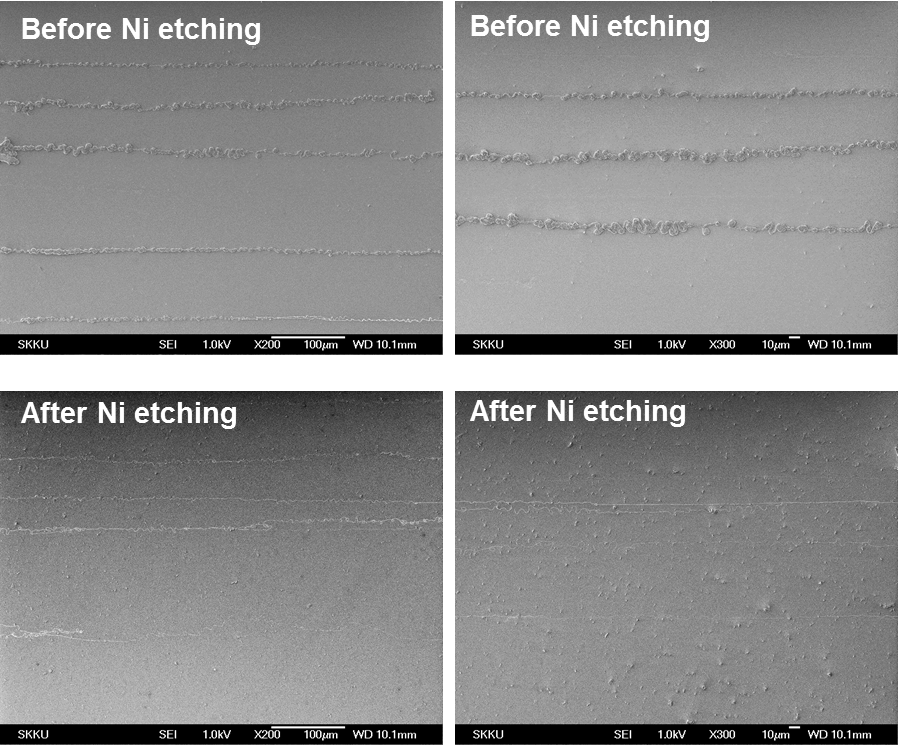


**Figure S6I** SEM images before and after Ni removal on m-SWCNT.

**S7. Source-drain pairs array for electrical characterization of sorted semiconducting and metallic SWCNT along the 1-cm-long SWCNT.**

**Figure S7I a,** Optical image of FET array fabricated on 1-cm-long SWCNT arrays. SWCNTs are grown from catalyst on the left edge of the wafer to the parallel right direction. **b,** Optical microscope image of magnified FET array of **a**. **c,** Optical image of the representative source-drain pair with channel gap of 10 μm. About 3~10 SWCNTs were contained in the 250 μm channel width.

**S8. On/off ratio of before and after sorting SWCNTs along the rows of electrodes array.**


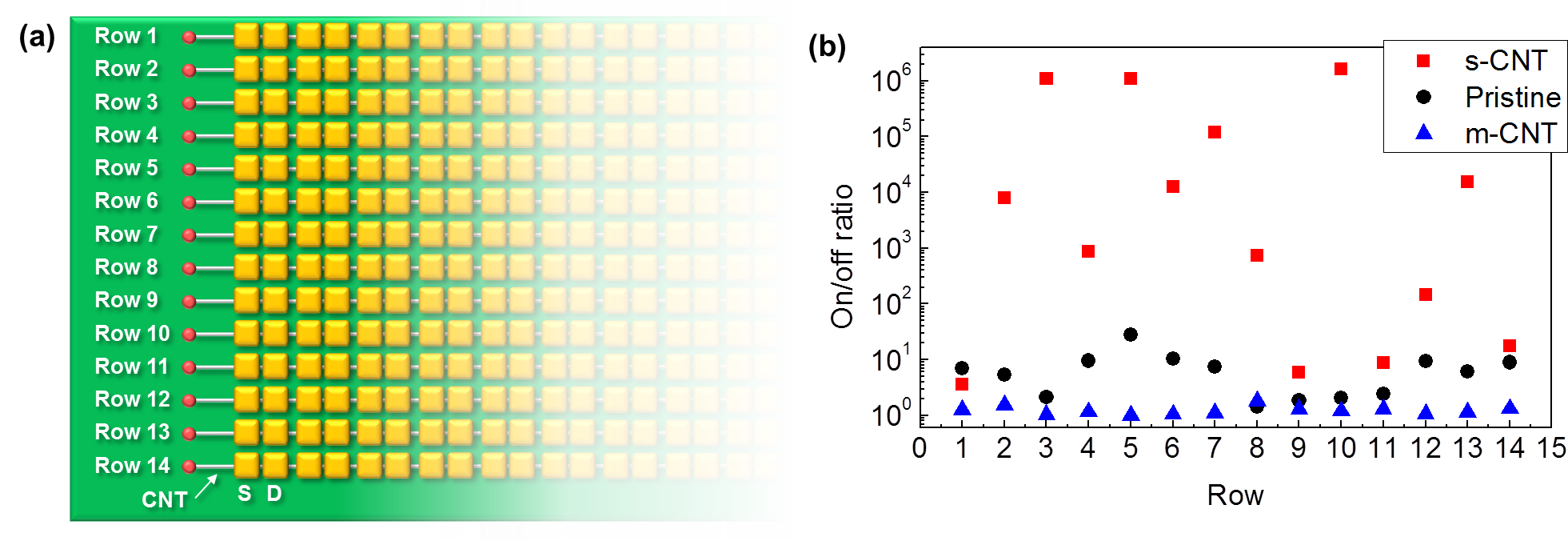


**Figure S8I** (a) Schematic layout of the electrodes array. (b) On/off ratio statistics of 14 rows of electrodes array.
